# Supplementary material for: Glacier retreat creating new Pacific salmon habitat in western North America
Source: Nat Commun. 2021 Dec 7;12:6816. doi: 10.1038/s41467-021-26897-2 (PMC8651712; doi:10.1038/s41467-021-26897-2)
Supplement: Supplementary file 1 — Supplementary Information [file 41467_2021_26897_MOESM1_ESM.pdf]

Supplementary information

Glacier retreat creating new Pacific salmon habitat in western North America

Kara J. Pitman[1*], Jonathan W. Moore[1], Matthias Huss[2,3,4], Matthew R. Sloat[5], Diane C. Whited[6], Tim J. Beechie[7], Rich Brenner[8], Eran W. Hood[9], Alexander M. Milner[10,11], George R. Pess[12], Gordan H. Reeves[13], Daniel E. Schindler[14]

[1]Earth to Ocean Research Group, Simon Fraser University, Burnaby, British Columbia, Canada. [2]Laboratory of Hydraulics, Hydrology and Glaciology (VAW), ETH Zurich, Zurich, Switzerland. [3]Department of Geosciences, University of Fribourg, Fribourg, Switzerland. [4]Swiss Federal Institute for Forest, Snow and Landscape Research (WSL), Birmensdorf, Switzerland. [5]Wild Salmon Center, Portland, OR, USA. [6]Flathead Lake Biological Station, University of Montana, Polson, USA. [7]Watershed Program, Fish Ecology Division, Northwest Fisheries Science Center, NOAA Fisheries, Seattle, Washington, USA. [8]Alaska Department of Fish and Game, Division of Commercial Fisheries, Juneau, AK, USA. [9]Program on the Environment, University of Alaska Southeast, Juneau, USA. [10]School of Geography, Earth and Environmental Science, University of Birmingham, Edgbaston, Birmingham, UK. [11]Institute of Arctic Biology, University of Alaska, Fairbanks, Alaska, 99775, USA. [12]Fish Ecology Division, Northwest Fisheries Science Center, National Marine Fisheries Service, NOAA Fisheries, Seattle, Washington, USA. [13]USDA Forest Service, Pacific Northwest Research Station, Corvallis, USA. [14]School of Aquatic and Fishery Sciences, University of Washington, Seattle, USA.

*Corresponding author: karapitman@gmail.com

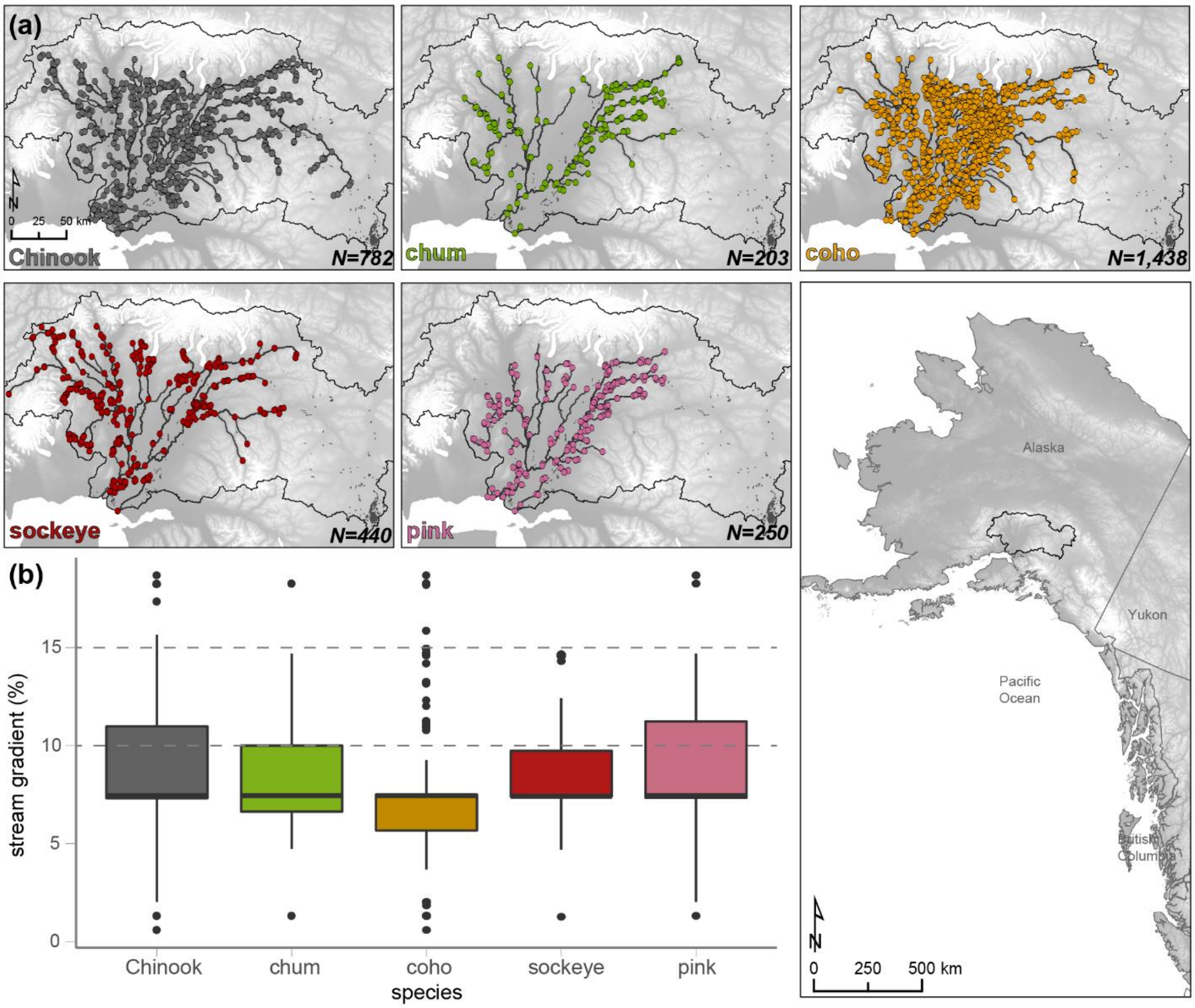

Supplementary Fig. 1: Migration stream gradient thresholds for each Pacific salmon species in the Susitna River, Alaska. **A** points represent individual Pacific salmon presence organized by species, obtained from the Anadromous Waters Catalogue (AWC; www.adfg.alaska.gov/sf/SARR/AWC). **B** Box plots representing the maximum stream gradient value crossed between river outlet and individual salmon observation for each Pacific salmon species. Box plots indicate the median (middle line), whiskers as max value (top) and min value (bottom), and quartiles at 25 percentile (lower) and 75 percentile (upper). We focus on the upper quartiles, and it can be concluded that for each species of salmon, with the exception of coho, 75% of the salmon observations cross a maximum gradient threshold of at least 10%. Dotted lines represent the 10% and 15% stream gradient thresholds. Salmon observation data are open source from the AWC. N refers to the number of salmon observations for each species shown in **A**. Source data are provided as a Source Data file.

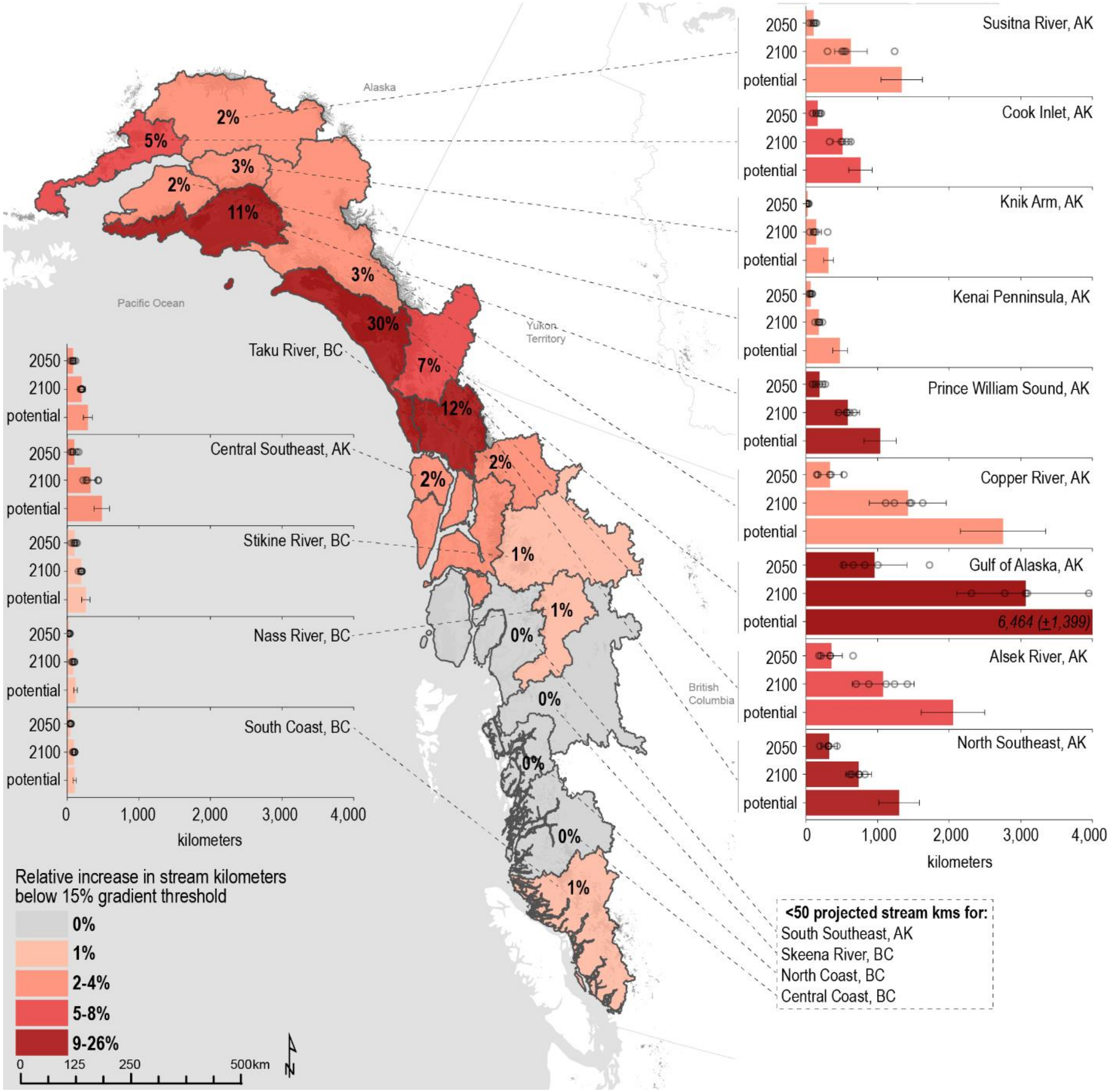

Supplementary Fig. 2: Projected future salmon-accessible stream kms with <15% stream gradient migration threshold. Map showing projected percent increase in future salmon-accessible stream kms, below a 10% stream gradient threshold for 2100, relative to present-day stream kms summed for each of the 18 sub-regions. Colors are defined in map. Glacier retreat projections, in response to five GCMs with RCP4.5 emission scenario, are used as an ensemble-mean. Bar plots representing ensemble-mean projected salmon-accessible stream kms with <15% stream gradient threshold for the years 2050, 2100, and potential complete deglaciation (i.e., once glaciers have retreated completely from the landscape) for each of the 18 sub-regions having >0% increase in future salmon-accessible stream kms. Projections are computed from 10-year averages centred around 2050 and 2100. Colors reflect percent increase values shown in map. Error bars correspond to ensemble-mean ± one standard deviation and originate from: GCM projections (RCP4.5, for 2050 and 2100), ice thickness estimates, and stream segment

length (see Methods section “Uncertainty estimates’). Points represent projections for the individual GCMs for 2050 and 2100 (N=5). Projected future salmon-accessible stream kms (± one standard deviation) are presented in the Source Data File

Supplementary Table 1: Metadata for the 18 sub-regions

Sub-region	Watershed size (km^2)	Total number of glaciers	Total glacier area (km^2)	Stream network size (km)	Number of accessible glaciers*		Accessible glacier area (km^2)	
					10%	15%	10%	15%
Susitna River, AK	53,366	1,278	4,413	34,336	24	51	2,598	3,198
Cook Inlet, AK	20,366	1,323	3,307	10,115	37	53	1,942	2,150
Knik Arm, AK	12,629	730	1,954	4,961	4	10	792	1,126
Kenai Peninsula, AK	19,054	827	1,691	11,729	13	21	820	973
Prince William Sound, AK	32,204	1,877	5,800	5,236	47	76	2,508	4,208
Copper River, AK	68,035	3,312	13,327	48,048	60	93	7,272	11,473
Gulf of Alaska, AK	34,033	1,251	17,678	10,016	38	55	15,459	16,929
Alsek River, AK	30,115	1,457	6,631	16,290	32	71	2,715	5,790
North southeast, AK	23,432	2,284	5,908	6,191	31	54	3,722	4,461
Taku River, BC	19,445	723	2,285	10,378	8	17	1,242	1,407
Central southeast, AK	44,427	1,501	4,092	19,785	8	18	1,680	2,299
Stikine River, BC	51,136	2,307	3,591	30,361	9	26	479	812
Nass River, BC	21,482	926	1,290	11,097	0	9	0	242
South southeast, AK	44,571	1,555	1,698	16,543	0	13	0	196
Skeena River, AK	54,404	1,282	820	35,636	1	5	3	11
North Coast, BC	16,381	702	358	6,387	1	3	2	2
Central Coast, BC	36,294	1,874	1,806	16,277	0	18	0	71
South Coast, BC	41,771	2,421	4,640	12,557	2	10	510	690
**Study region**	**623,152**	**27,630**	**81,289**	**305,943**	**315**	**603**	**41,743**	**56,039**

*Number of accessible glaciers were derived from RCP 4.5 projections for the year 2100.

Supplementary Table 2: Stream gradient representing habitat suitability for spawning and rearing for different Pacific salmon species including referenced literature.

species	value	location	life phase	resource
coho	0 – 4%	South Fork Stillaguamish River, USA	spawning and rearing	[79]
coho	0 – 4%	Skagit River, WA	spawning	[28]
coho	0 – 4.8%	Washington State, USA	rearing	[80]
coho	0 – 3%	Regional	rearing	[31]
coho	0 – 7%[1]	western Oregon	rearing	[26]
coho	0 – 7%	Pacific Northwest	spawning and rearing	[27]
coho	0 – 4.5%	southeast Alaska	spawning	[74]
chum	0 – 3%	Pacific Northwest	spawning and rearing	[27]
chum	0 – 4.5%	southeast Alaska	spawning	[74]
pink	0 – 4.5%	southeast Alaska	spawning	[74]
sockeye	0 – 7%	Pacific Northwest	spawning and rearing	[27]
Chinook	0 – 1.5%	Puget Sound	rearing	[25]
Chinook	0 – 7%	Pacific Northwest	spawning and rearing	[27]
Chinook	<4%[2]	Copper River, AK	spawning and rearing	[29]
steelhead	0 – 4%	South Fork Stillaguamish River, USA	spawning and rearing	[79]
steelhead	0 – 4.8%	Washington State, USA	rearing	[80]
steelhead	0.5 – 7%[3]	Puget Sound	rearing	[25]
steelhead	2 – 3%[4]	western Oregon	rearing	[26]
steelhead	0 – 8%	Pacific Northwest	spawning and rearing	[27]

[1]rear mostly in low gradients and decrease in density as slope increases, nothing upstream of 7%
[2]Highest intrinsic potential between 0.5 – 1.5%.
[3]densities remained high as gradients increased >4%. Low density at gradients <0.5%, increasing as gradients rose to ~4%.
[4]2 – 3% are optimal, no use upstream of reaches with gradients exceeding 10%.

Supplementary Table 3: Total future salmon-accessible streams for the 18 sub-regions when considering the 10% and 15% stream gradient thresholds using two emission scenarios (RCP4.5, RCP8.5) for the years 2050, 2100, and potential complete deglaciation.

Sub-region	Total kms (RCP 4.5) 2050	Total kms (RCP 4.5) 2100	Total kms (RCP 8.5) 2050	Total kms (RCP 8.5) 2100	Complete Deglaciation
**10%**					
Susitna River, AK	87 (±32)	381 (±131)	80 (±31)	379 (±103)	675 (±146)
Cook Inlet, AK	136 (±42)	319 (±73)	132 (±46)	341 (±76)	423 (±92)
Knik Arm, AK	18 (±10)	102 (±61)	17 (±9)	106 (±41)	212 (±46)
Kenai Peninsula, AK	44 (±16)	95 (±22)	44 (±18)	102 (±22)	104 (±23)
Prince William Sound, AK	120 (±45)	272 (±67)	116 (±43)	333 (±75)	429 (±93)
Copper River, AK	305 (±151)	1,064 (±344)	290 (±138)	1,238 (±327)	1,713 (±371)
Gulf of Alaska, AK	909 (±401)	2,622 (±764)	899 (±332)	3,241 (±792)	4,423 (±958)
Alsek River, AK	221 (±79)	530 (±122)	209 (±69)	604 (±133)	731 (±158)
North southeast, AK	214 (±61)	384 (±89)	207 (±63)	445 (±99)	546 (±118)
Taku River, BC	41 (±12)	81 (±21)	41 (±11)	92 (±21)	120 (±26)
Central southeast, AK	58 (±22)	166 (±45)	54 (±18)	204 (±49)	243 (±53)
Stikine River, BC	54 (±16)	100 (±23)	52 (±15)	107 (±23)	111 (±24)
Nass River, BC	0 (±0)	0 (±0)	0 (±0)	0 (±0)	0 (±0)
South southeast, AK	0 (±0)	0 (±0)	0 (±0)	0 (±0)	0 (±0)
Skeena River, AK	3 (±1)	3 (±1)	3 (±1)	3 (±0)	3 (±1)
North Coast, BC	2 (±0)	2 (±0)	2 (±0)	2 (±0)	2 (±0)
Central Coast, BC	0 (±0)	0 (±0)	0 (±0)	0 (±0)	0 (±0)
South Coast, BC	20 (±5)	25 (±5)	19 (±5)	25 (±5)	25 (±5)
**Study region**	**2,231 (±844)**	**6,146 (±1,619)**	**2,166 (±801)**	**7,224 (±1,693)**	**9,759 (±2,113)**
**15%**					
Susitna River, AK	106 (±44)	628 (±228)	96 (±40)	592 (±158)	1,336 (±289)
Cook Inlet, AK	165 (±61)	510 (±129)	163 (±68)	567 (±134)	763 (±165)
Knik Arm, AK	25 (±15)	144 (±72)	24 (±13)	151 (±54)	316 (±68)
Kenai Peninsula, AK	68 (±26)	181 (±49)	67 (±30)	241 (±60)	475 (±103)
Prince William, AK	190 (±81)	585 (±163)	188 (±77)	789 (±184)	1,038 (±225)
Copper River, AK	338 (±165)	1,423 (±537)	313 (±146)	1,694 (±511)	2,752 (±596)
Gulf of Alaska, AK	958 (±450)	3,067 (±960)	949 (±362)	3,873 (±1,022)	6,464 (±1,399)
Alsek River, AK	355 (±153)	1,078 (±432)	326 (±120)	1,301 (±351)	2,053 (±444)
North southeast, AK	324 (±113)	734 (±181)	316 (±101)	913 (±221)	1,301 (±282)
Taku River, BC	83 (±32)	203 (±51)	81 (±27)	244 (±55)	290 (±63)
Central southeast, AK	99 (±48)	326 (±86)	92 (±36)	400 (±91)	484 (±105)
Stikine River, BC	104 (±35)	196 (±46)	101 (±34)	221 (±49)	261 (±57)
Nass River, BC	27 (±11)	86 (±20)	25 (±10)	101 (±22)	117 (±25)
South southeast, AK	0 (±0)	0 (±0)	0 (±0)	0 (±0)	53 (±11)
Skeena River, AK	9 (±2)	9 (±2)	9 (±2)	9 (±2)	10 (±2)
North Coast, BC	2 (±0)	2 (±0)	2 (±0)	2 (±0)	3 (±1)
Central Coast, BC	29 (±7)	29 (±6)	29 (±7)	29 (±7)	35 (±8)
South Coast, BC	46 (±13)	95 (±22)	45 (±12)	105 (±23)	105 (±23)
**Study region**	**2,929 (±1,230)**	**9,296 (±2,740)**	**2,827 (±1,116)**	**11,231 (±2,826)**	**17857 (±3,866)**

Uncertainty corresponds to ± one standard deviation and originate from: GCM projections (RCP4.5 and 8.5, for 2050 and 2100), ice thickness estimates, and stream segment length (see Methods section "Uncertainty estimates').

Supplementary Table 4: Total future spawning and rearing habitat kilometers for the 18 sub-regions when considering 10% stream gradient thresholds, 0-2% spawning and rearing habitat and 15% stream gradient thresholds, 0-4% spawning and rearing habitat for two emission scenarios (RCP4.5, RCP8.5) for the years 2050, 2100.

**Sub-region**	**Total kms (RCP 4.5)**		**Total kms (RCP 8.5)**	
	**2050**	**2100**	**2050**	**2100**
**10% stream gradient migration threshold, 0-2% spawning and rearing habitat**				
Susitna River, AK	35 (±14)	124 (±51)	32 (±11)	122 (±34)
Cook Inlet, AK	31 (±9)	86 (±23)	29 (±10)	93 (±22)
Knik Arm, AK	8 (±3)	37 (±28)	7 (±3)	37 (±11)
Kenai Peninsula, AK	14 (±6)	25 (±6)	14 (±5)	27 (±6)
Prince William Sound, AK	33 (±12)	71 (±17)	34 (±13)	82 (±19)
Copper River, AK	132 (±57)	408 (±105)	128 (±49)	471 (±130)
Gulf of Alaska, AK	255 (±118)	757 (±279)	250 (±86)	958 (±266)
Alsek River, AK	78 (±29)	160 (±43)	75 (±33)	177 (±43)
North southeast, AK	66 (±19)	128 (±33)	65 (±18)	146 (±33)
Taku River, BC	12 (±4)	25 (±6)	12 (±4)	29 (±7)
Central southeast, AK	19 (±8)	61 (±21)	18 (±7)	79 (±23)
Stikine River, BC	22 (±7)	42 (±10)	21 (±6)	43 (±10)
Nass River, BC	0 (±0)	0 (±0)	0 (±0)	0 (±0)
South southeast, AK	0 (±0)	0 (±0)	0 (±0)	0 (±0)
Skeena River, AK	1 (±0)	1 (±0)	1 (±0)	1 (±0)
North Coast, BC	0 (±0)	0 (±0)	0 (±0)	0 (±0)
Central Coast, BC	0 (±0)	0 (±0)	0 (±0)	0 (±0)
South Coast, BC	5 (±2)	5 (±0)	5 (±1)	5 (±1)
**Study region**	**710 (±263)**	**1,930 (±569)**	**691 (±234)**	**2,270 (±551)**
**15% stream gradient migration threshold, 0-4% spawning and rearing habitat**				
Susitna River, AK	42 (±18)	215 (±100)	38 (±18)	202 (±57)
Cook Inlet, AK	46 (±17)	150 (±42)	44 (±21)	165 (±42)
Knik Arm, AK	10 (±5)	58 (±38)	9 (±4)	58 (±26)
Kenai Peninsula, AK	22 (±9)	62 (±17)	21 (±12)	84 (±27)
Prince William Sound, AK	65 (±25)	197 (±51)	66 (±28)	255 (±64)
Copper River, AK	138 (±71)	550 (±144)	132 (±52)	657 (±177)
Gulf of Alaska, AK	287 (±143)	929 (±310)	280 (±109)	1,201 (±362)
Alsek River, AK	135 (±87)	384 (±138)	127 (±63)	461 (±145)
North southeast, AK	111 (±41)	275 (±73)	109 (±31)	349 (±87)
Taku River, BC	35 (±16)	78 (±20)	34 (±14)	94 (±23)
Central southeast, AK	40 (±19)	126 (±49)	38 (±16)	156 (±40)
Stikine River, BC	44 (±16)	83 (±20)	43 (±15)	91 (±21)
Nass River, BC	11 (±4)	29 (±8)	10 (±4)	32 (±8)
South southeast, AK	0 (±0)	0 (±0)	0 (±0)	0 (±0)
Skeena River, AK	1 (±0)	1 (±0)	1 (±1)	1 (±0)
North Coast, BC	0 (±0)	0 (±0)	0 (±0)	0 (±0)
Central Coast, BC	3 (±1)	3 (±1)	3 (±1)	3 (±1)
South Coast, BC	17 (±5)	37 (±9)	17 (±5)	39 (±9)
**Study region**	**1,008 (±423)**	**3,177 (±981)**	**973 (±368)**	**3,848 (±1,036)**

Uncertainty corresponds to ± one standard deviation and originate from: GCM projections, ice thickness estimates, and stream segment length (see Methods section "Uncertainty estimates').

Supplementary Table 5: Number of juveniles per km for coho salmon including referenced literature.

species	value	location	resource
coho	600 juveniles/km	Skagit River, WA	[28,68]
coho	2,732 juveniles/km	Big Qualicum River, BC	[81]
coho	1,367 juveniles/km	glacially fed streams	[82]
coho	250 juveniles/km	Baker River, WA	[83]
coho	457-1,476 juveniles/km	Regional	[31]

Supplementary Table 6 | Total kms by stream order once the glaciers have reached potential complete deglaciation.

	<10% stream gradient					<15% stream gradient				
	stream order									
	1st	2nd	3rd	4th	5th	1st	2nd	3rd	4th	5th
Susitna River, AK	404	151	93	15	0	723	269	196	18	0
Cook Inlet, AK	268	94	50	0	0	374	177	100	0	0
Knik Arm, AK	127	38	44	0	0	155	66	65	0	0
Kenai Peninsula, AK	58	29	13	0	0	208	107	39	32	22
Prince William Sound, AK	265	105	48	0	0	486	245	137	12	0
Copper River, AK	1,008	452	219	12	0	1,406	678	396	37	0
Gulf of Alaska, AK	2,918	954	299	179	52	3,883	1,375	527	320	62
Alsek River, AK	493	171	57	0	0	1,077	471	235	94	4
North Southeast, AK	297	167	56	16	0	579	330	203	46	0
Taku River, BC	69	41	8	0	0	125	102	29	0	0
Central Southeast, AK	121	61	32	26	0	219	123	64	26	0
Stikine River, BC	56	32	21	0	0	105	88	31	0	0
Nass River, BC	0	0	0	0	0	45	26	24	0	0
South Southeast, AK	0	0	0	0	0	26	18	0	0	0
Skeena River, BC	2	1	0	0	0	5	1	0	0	0
North Coast, BC	2	0	0	0	0	2	0	0	0	0
Central Coast, BC	0	0	0	0	0	18	5	0	0	0
South Coast, BC	18	7	0	0	0	44	30	18	0	0
**Study region**	**6,106**	**2,303**	**940**	**248**	**52**	**9,480**	**4,111**	**2,064**	**585**	**88**

Supplementary Table 7 | Total number (and area) of glaciers that salmon can access modelled from present day streams based on the 10% stream gradient threshold using four different stream segment lengths for two sample sub-regions, North Southeast, AK, and Taku River, BC. Bolded is the ~500 m segment length used in this analysis.

	**North Southeast, AK**		**Taku River, BC**	
	**number of glaciers**	**area (HA)**	**number of glaciers**	**area (HA)**
250m	27	3593.7	8	1241.9
400m	32	3889.0	8	1241.9
**500m**	**31**	**3722.0**	**8**	1241.9
600m	33	3821.5	8	1241.9
750m	35	3857.5	8	1241.9

Supplementary Table 8 | Total future salmon-accessible stream kilometres using the 10% stream gradient threshold for two different segment length scenarios, ~250 m and ~750 m for once the glaciers have reached potential complete deglaciation summed by sub-region. Included is the % change from the ~500 m segment length used in our analysis.

	500 m	250 m	% change	750 m	% change
Susitna River, AK	675	596	-12	707	5
Cook Inlet, AK	423	387	-8	459	9
Knik Arm, AK	212	183	-14	229	8
Kenai Peninsula, AK	104	76	-27	102	-2
Prince William Sound, AK	429	301	-30	424	-1
Copper River, AK	1,713	1,284	-25	1,878	10
Gulf of Alaska, AK	4,423	4,134	-7	4,932	11
Alsek River, AK	721	578	-21	807	10
North Southeast, AK	546	433	-21	662	21
Taku River, BC	120	94	-22	124	3
Central Southeast, AK	243	204	-16	367	51
Stikine River, BC	111	91	-18	111	0
Nass River, BC	0	0	0	0	0
South Southeast, AK	0	0	0	0	0
Skeena River, BC	3	3	3	3	14
North Coast, BC	2	2	-2	2	-10
Central Coast, BC	0	0	0	0	0
South Coast, BC	25	24	-6	25	-1
**Study region**	**9,750**	**8,390**	**-14**	**10,832**	**11**
