# Supplementary material for: Glacier retreat creating new Pacific salmon habitat in western North America
Source: Nat Commun. 2021 Dec 7;12:6816. doi: 10.1038/s41467-021-26897-2 (PMC8651712; doi:10.1038/s41467-021-26897-2)
Supplement: Supplementary file 3 — Reporting Summary [file 41467_2021_26897_MOESM3_ESM.pdf]

nature research

Corresponding author(s): Kara J Pitman

Last updated by author(s): Oct 20, 2021

# Reporting Summary

Nature Research wishes to improve the reproducibility of the work that we publish. This form provides structure for consistency and transparency in reporting. For further information on Nature Research policies, see our Editorial Policies and the Editorial Policy Checklist.

## Statistics

For all statistical analyses, confirm that the following items are present in the figure legend, table legend, main text, or Methods section.

| n/a | Confirmed | |
|---|---|---|
| ☐ | ☒ | The exact sample size (*n*) for each experimental group/condition, given as a discrete number and unit of measurement |
| ☒ | ☐ | A statement on whether measurements were taken from distinct samples or whether the same sample was measured repeatedly |
| ☒ | ☐ | The statistical test(s) used AND whether they are one- or two-sided<br>*Only common tests should be described solely by name; describe more complex techniques in the Methods section.* |
| ☒ | ☐ | A description of all covariates tested |
| ☒ | ☐ | A description of any assumptions or corrections, such as tests of normality and adjustment for multiple comparisons |
| ☐ | ☒ | A full description of the statistical parameters including central tendency (e.g. means) or other basic estimates (e.g. regression coefficient) AND variation (e.g. standard deviation) or associated estimates of uncertainty (e.g. confidence intervals) |
| ☒ | ☐ | For null hypothesis testing, the test statistic (e.g. *F*, *t*, *r*) with confidence intervals, effect sizes, degrees of freedom and *P* value noted<br>*Give P values as exact values whenever suitable.* |
| ☒ | ☐ | For Bayesian analysis, information on the choice of priors and Markov chain Monte Carlo settings |
| ☒ | ☐ | For hierarchical and complex designs, identification of the appropriate level for tests and full reporting of outcomes |
| ☒ | ☐ | Estimates of effect sizes (e.g. Cohen's *d*, Pearson's *r*), indicating how they were calculated |

*Our web collection on statistics for biologists contains articles on many of the points above.*

## Software and code

Policy information about availability of computer code

| | |
|---|---|
| Data collection | No software was used for data collection |
| Data analysis | Data analysis for this study was conducted using both ArcGIS 10.6 and QGIS 2.18.27, and RivEX (ver 10.27) for spatial analysis. We used R software (rstudio: 1.4.1103-4, R:'Mirrors') to summarize data. We used the Global Glacier Evolution Model (GloGEM) for determining timing of glacier retreat and ice thickness data. |

For manuscripts utilizing custom algorithms or software that are central to the research but not yet described in published literature, software must be made available to editors and reviewers. We strongly encourage code deposition in a community repository (e.g. GitHub). See the Nature Research guidelines for submitting code & software for further information.

## Data

Policy information about availability of data

All manuscripts must include a data availability statement. This statement should provide the following information, where applicable:
- Accession codes, unique identifiers, or web links for publicly available datasets
- A list of figures that have associated raw data
- A description of any restrictions on data availability

The new accessible stream kilometer data generated in this study have been deposited in a Zenodo database (here we reference the database, as presented in the references). All other spatial datasets were obtained from open access sources: DEMs used in this analysis can be downloaded from NASA (https://asterweb.jpl.nasa.gov/gdem.asp); watershed boundary data for the USA are available at USGS (https://www.usgs.gov), and from at Freshwater atlas of BC for British Columbia (https://www2.gov.bc.ca/gov); glacier outline data can be found at the Glacier Inventory v6.0 (https://www.glims.org/RGI/); Pacific salmon presence data are available from the Anadromous Waters Catalogue (AWC; www.adfg.alaska.gov/sf/SARR/AWC).

# Field-specific reporting

Please select the one below that is the best fit for your research. If you are not sure, read the appropriate sections before making your selection.

☐ Life sciences ☐ Behavioural & social sciences ☒ Ecological, evolutionary & environmental sciences

For a reference copy of the document with all sections, see nature.com/documents/nr-reporting-summary-flat.pdf

# Ecological, evolutionary & environmental sciences study design

All studies must disclose on these points even when the disclosure is negative.

| | |
|---|---|
| Study description | Our study projects the extent of future gains in Pacific salmon freshwater habitat throughout the Pacific mountain ranges of western North America. We linked a model of glacier mass change for almost 600 glaciers, forced by five different Global Climate Models, with a simple model of salmon stream habitat potential across a 623,000 km2 study region. We then quantified where throughout our study region would experience gains in salmon habitat for the years 2050 and 2100. The design structure is factorial. |
| Research sample | DEMs used in this analysis can be downloaded from NASA (https://asterweb.jpl.nasa.gov/gdem.asp) or Shuttle Rada Topography Mission (SRTM) with a spatial resolution of ~ 30m and vertical resolution of ~¬¬+ 5 m. Watershed boundary data for the USA were sourced from USGS (https://www.usgs.gov) and from the Freshwater atlas of BC for British Columbia (https://www2.gov.bc.ca/gov). Glacier outline data was obtained from the Glacier Inventory v6.0 (https://www.glims.org/RGI/). Pacific salmon presence data were available from the Anadromous Waters Catalogue (AWC; www.adfg.alaska.gov/sf/SARR/AWC). Present-day rivers were represented by building a synthetic stream network using ArcGIS 10.6 and previously mentioned DEMs. Ice thickness distribution was calculated at a grid resolution of 25 to 200 m (depending on glacier area) using a simple dynamic model that considers glacier mass turnover and ice flow mechanics, and by inverting the glaciers' surface topography. For Glacier retreat data, we used the Global Glacier Evolution Model (GloGEM), which computes glacier mass balance and associated geometry changes for each individual glacier in the study region. GloGEM is forced with temperturea and precipitation time series from an ensemble of five Global Climate Models (GCMs). The five GCM included: CanESM2, CSIRO-Mk3-6-0, GFDL-CM3, MIROC-ESM, and MPI-ESM-LR, and we presented projections from Representative Concentration Pathways (RCPs): RCP4.5 and RCP8.5, which correspond to plausible scenarios for the rate of change in the concentration of atmospheres CO2 and other greenhouse gas emissions. |
| Sampling strategy | We used spatial analysis to determine our findings. Therefore, did not use any statistical methods to predetermine sample size. Our sample size was determined by the total number of glaciers within the study region (n = ~300), and the number of subregions (n= 18) within our study region. We considered glacier retreat and ice thickness for ~300 glaciers within the Pacific mountain ranges of North American. We summarized these findings by 18 subregions, which were selected based on their watershed delineations, and where there were known salmon present in these watersheds. All watersheds that contained less than 1.5% glacier cover were included in our analysis. We ran 5 GCM model projections to determine timing of glacier retreat. These 5 GCM were chosen based on previous literatures assessments of most suitable projections for western North America. |
| Data collection | All of our data were spatial datasets obtained from open access sources: DEMs were downloaded from NASA (https://asterweb.jpl.nasa.gov/gdem.asp); watershed boundary data for the USA were downloaded from USGS (https://www.usgs.gov), and from at Freshwater atlas of BC for British Columbia (https://www2.gov.bc.ca/gov); glacier outline data were obtained from the Glacier Inventory v6.0 (https://www.glims.org/RGI/); Pacific salmon presence data were obtained from the Anadromous Waters Catalogue (AWC; www.adfg.alaska.gov/sf/SARR/AWC). |
| Timing and spatial scale | There are no start and stop dates associated with the data collection as the data were sourced from Open sources. However, the glacier outline data refer roughly to the years 2009 + 2 for Alaska, and 2004 + 5 for Western Canada. All data obtained from open sources range from southern British Columbia, Canada to Alaska, USA. |
| Data exclusions | There were no data excluded from the analysis. |
| Reproducibility | We did not conduct any experimental designs, therefore do not have reproducibility to verify. We did run sensitivity analyses on stream segment lengths and total number of glaciers selected in our analyses. This was conducted to determine the error associated with our findings. In our analysis, we determined the total number of glaciers accessible as well as future salmon-accessible stream kilometers for salmon by using stream segment lengths of ~500m, but also ran a sensitivity analysis using segment lengths of 250m, 400m, 600m and 750m for glacier selection and 250m and 750m segment length for future stream kilometers. |
| Randomization | We did not need to consider randomization given that we did not conduct a statistical analysis but used spatial analysis instead. We did assess future stream kilometer gains for salmon across 18 sub regions, which were determined using the delineation of watershed boundaries. |
| Blinding | We did not need to consider blinding, as we did not conduct our analysis using an experimental design. |

Did the study involve field work? ☐ Yes ☒ No

# Reporting for specific materials, systems and methods

We require information from authors about some types of materials, experimental systems and methods used in many studies. Here, indicate whether each material, system or method listed is relevant to your study. If you are not sure if a list item applies to your research, read the appropriate section before selecting a response.

## Materials & experimental systems

| n/a | Involved in the study |
| --- | --- |
| ☒ | ☐ Antibodies |
| ☒ | ☐ Eukaryotic cell lines |
| ☒ | ☐ Palaeontology and archaeology |
| ☒ | ☐ Animals and other organisms |
| ☒ | ☐ Human research participants |
| ☒ | ☐ Clinical data |
| ☒ | ☐ Dual use research of concern |

## Methods

| n/a | Involved in the study |
| --- | --- |
| ☒ | ☐ ChIP-seq |
| ☒ | ☐ Flow cytometry |
| ☒ | ☐ MRI-based neuroimaging |
